# A Computational Approach for Deciphering the Organization of Glycosaminoglycans

**DOI:** 10.1371/journal.pone.0009389

**Published:** 2010-02-23

**Authors:** Jean L. Spencer, Joel A. Bernanke, Jo Ann Buczek-Thomas, Matthew A. Nugent

**Affiliations:** 1 Department of Biochemistry, Boston University School of Medicine, Boston, Massachusetts, United States of America; 2 Department of Ophthalmology, Boston University School of Medicine, Boston, Massachusetts, United States of America; 3 Department of Biomedical Engineering, Boston University, Boston, Massachusetts, United States of America; Massachusetts Institute of Technology, United States of America

## Abstract

**Background:**

Increasing evidence has revealed important roles for complex glycans as mediators of normal and pathological processes. Glycosaminoglycans are a class of glycans that bind and regulate the function of a wide array of proteins at the cell-extracellular matrix interface. The specific sequence and chemical organization of these polymers likely define function; however, identification of the structure-function relationships of glycosaminoglycans has been met with challenges associated with the unique level of complexity and the nontemplate-driven biosynthesis of these biopolymers.

**Methodology/Principal Findings:**

To address these challenges, we have devised a computational approach to predict fine structure and patterns of domain organization of the specific glycosaminoglycan, heparan sulfate (HS). Using chemical composition data obtained after complete and partial digestion of mixtures of HS chains with specific degradative enzymes, the computational analysis produces populations of theoretical HS chains with structures that meet both biosynthesis and enzyme degradation rules. The model performs these operations through a modular format consisting of input/output sections and three routines called chainmaker, chainbreaker, and chainsorter. We applied this methodology to analyze HS preparations isolated from pulmonary fibroblasts and epithelial cells. Significant differences in the general organization of these two HS preparations were observed, with HS from epithelial cells having a greater frequency of highly sulfated domains. Epithelial HS also showed a higher density of specific HS domains that have been associated with inhibition of neutrophil elastase. Experimental analysis of elastase inhibition was consistent with the model predictions and demonstrated that HS from epithelial cells had greater inhibitory activity than HS from fibroblasts.

**Conclusions/Significance:**

This model establishes the conceptual framework for a new class of computational tools to use to assess patterns of domain organization within glycosaminoglycans. These tools will provide a means to consider high-level chain organization in deciphering the structure-function relationships of polysaccharides in biology.

## Introduction

Complex glycans such as glycosaminoglycans (GAGs) are rapidly becoming appreciated as major regulators of cell function throughout the animal kingdom [1]–[3]. In particular, the GAG chains of proteoglycans have been shown to play important roles in mediating cell-extracellular matrix interactions, extracellular matrix structure and function, and cell-cell communication principally through the ability of GAGs to bind to a wide range of proteins [4], [5]. Heparan sulfate (HS), the most structurally diverse GAG class, has been implicated in countless normal and pathological biological processes [1], [3], [6], [7]. HS is a linear polysaccharide composed of repeating disaccharide units of hexuronic acid (D-glucuronic acid or L-iduronic acid) and D-glucosamine (*N*-unsubstituted, *N*-acetylated, or *N*-sulfated) with varying degrees of *O*-sulfation. The disaccharides are clustered in alternating domains of short segments of highly sulfated disaccharides (S-domains) and longer segments of predominantly unmodified disaccharides [1], [8], [9]. Since the discovery that a rare sequence of five sugars in heparin is responsible for the binding and functional modulation of antithrombin III [10], substantial effort has focused on specifying the sequences for other HS-protein interactions.

Although significant advances in the analytical methodology for HS sequencing have been made in recent years [5], [11]–[15], the current level of technology still lacks the capability to “sequence” HS chains longer than a decasaccharide. Instead, typical HS chains (25–200 disaccharides) are degraded with chemicals and enzymes of known specificity into a population of smaller segments that are separated by size and then purified and sequenced. Successful sequencing of small oligosaccharides containing ten or less sugars has been accomplished by a variety of techniques ranging from gel electrophoresis and chromatography to mass spectrometry (MS) and nuclear magnetic resonance (NMR) spectroscopy.

In a strategy called integral glycan sequencing (IGS), oligosaccharides are labeled on the reducing end with a fluorescent tag, partially cleaved with nitrous acid (low pH), and sequentially processed with different exoenzymes [16], [17]. The various stages of digested oligosaccharides are separated by polyacrylamide gel electrophoresis (PAGE), and the ladder-like pattern of fluorescent bands from a single run is used to directly read the saccharide sequence. Other methods follow a similar approach by labeling the oligosaccharides with different tags (e.g., radiolabels) and using a series of strong anion-exchange high-performance liquid chromatography (SAX-HPLC) steps to separate and identify the digests [18]–[20].

These relatively simple techniques of degradation and separation are frequently combined with more sophisticated instrumentation and software programs in sequencing strategies. For example, by integrating capillary electrophoresis (CE) with matrix-assisted laser desorption ionization MS (MALDI-MS), a list of all possible sequences for the oligosaccharide is generated using a scheme based upon a property-encoded nomenclature (PEN) [21], [22]. Subsequent chemical or enzymatic degradations of the oligosaccharide, followed by MALDI-MS identification of the fragments, allow convergence to a unique sequence on the list. In a related strategy using the PEN system, CE is paired with NMR spectroscopy to deduce the oligosaccharide sequence by combining complementary information on linkages between adjacent monosaccharides [23]. NMR data are acquired from a single series of one-dimensional (1D) proton and two-dimensional (2D) correlation spectroscopy (COSY)/total correlation spectroscopy (TOCSY) experiments using the intact oligosaccharide without need for further degradation. Another sequencing strategy integrates electrospray ionization MS (ESI-MS) of the intact oligosaccharide with ESI-MS and tandem MS (MS*^n^*) of the exhaustively digested oligosaccharide through a software application called the heparin oligosaccharide sequencing tool (HOST) to generate all possible sequences [24]. These sequences are then fragmented theoretically by the program, and product ions are compared with experimental fragments from MS*^n^* of the oligosaccharide to determine the best match.

Despite the continuing development of these analytical methods, the reality of the situation is that only short oligosaccharides can be fully sequenced, and the prospect of unmasking the structure of intact HS chains remains a formidable challenge. The original success of the heparin-antithrombin binding model, however, is becoming somewhat tempered by the recognition that the presence of distinct protein-specific saccharide sequences is more likely the exception than the rule [3], [5], [9], [12]. Although studies indicate that particular sulfated residues are required or more preferred in certain situations [25], protein binding may ultimately depend on the ability to properly position these residues with respect to complementary regions on the protein surface. While flexibility of the iduronic acid ring enhances local fit between binding partners, on a larger scale, this feature has minimal influence on the orientation of the chain [26]. Instead, the overall flexibility of the chain is defined by the spacing of the unmodified domains. Thus, variations in domain spacing and overall chain flexibility are likely to have dramatic effects on the potential of an HS chain to bind and modulate proteins [1], [26], [27].

The lack of an analytical capability to detect patterns of HS domain organization is a direct offshoot of the inability to fully sequence the chain, and the consequence of this deficiency has severely limited the understanding of HS structure-function relationships at a mechanistic level [28]. As a result, alternative approaches have been sought in an attempt to reveal information regarding the larger picture of domain organization. For example, an end-referencing approach was used to describe the domain structure of the first 36 disaccharides of an HS chain [29], and more recently, a method of selective lyase degradation was utilized to predict an average spacing of 16–18 disaccharides between highly sulfated domains [30]. However, there remains no generalized conceptual approach for exploring patterns of HS domain organization. Solutions to this challenge will require new and creative tools as alternative sources of information.

To address this need, a novel computational approach was developed for predicting the patterns of HS domain organization. Using experimental data from disaccharide analysis and selective heparin lyase digestion of HS samples, a computational routine was devised to generate populations of predicted HS chains that can be evaluated for the presence of general and specific structural properties. The generated chains are transformed into strings of user-defined domains and examined for patterns of domain organization. The model was tested by applying it to the analysis of two different samples of HS chains isolated from rat pulmonary fibroblast and epithelial cell cultures. The results show significant differences in the overall domain organization of these two samples as well as in the density of a specific structural motif proposed to be required for the inhibition of the inflammatory protease, neutrophil elastase. The findings herein indicate that this approach may provide an innovative tool for exploring the structural distinctions in various HS populations (i.e., from different tissues, organs, disease states, or stages of development) that could yield insight into the mechanisms of HS control of important biological processes.

## Materials and Methods

### Nomenclature

Disaccharides were designated by four alphanumeric characters called the disaccharide structural code [31]. For the relevant HS disaccharides of this study, the first two characters represent the hexuronic acid—a letter for the stereochemistry (D  =  unsaturated; G  =  glucuronic; I  =  iduronic) and a number for the location of *O*-sulfation (0 =  no sulfation; 2 = 2-*O*-sulfation), and the last two characters represent the glucosamine—a letter for the *N* substituent (H  =  free amine or *N*-unsubstituted; A  =  *N*-acetylated; S  =  *N*-sulfated) and a number for the location of *O*-sulfation (0 =  no sulfation; 6 = 6-*O*-sulfation).

### Materials

Heparin (porcine intestinal mucosa; 17–19 kDa), chondroitin-4-sulfate, and 1,9-dimethylmethylene blue were from Sigma-Aldrich (St. Louis, MO). Heparan sulfate (porcine intestinal mucosa; 8–10 kDa) was obtained from Neoparin (Alameda, CA). Human neutrophil elastase (human purulent sputum; 29.5 kDa) and elastin (bovine neck ligament; particle size <37 µm) were purchased from Elastin Products Company (Owensville, MO). Dulbecco's phosphate-buffered saline (PBS) without calcium and magnesium salts was ordered from Invitrogen (Carlsbad, CA).

### Chemical Structure Analysis of HS Preparations

Purified HS preparations were isolated from rat pulmonary fibroblasts [32] and epithelial cells [33] according to published methods [34]. Lyophilized samples of HS chains were sent to the Glycotechnology Core Resource at the University of California, San Diego (La Jolla, CA), for a series of heparin lyase digestions followed by HPLC profiling of the disaccharide products. All digestions were performed at 37°C overnight, and the resulting data are tabulated in Tables 1 and 2. The first set (Table 1) is the total disaccharide composition of each sample after exhaustive digestion with heparin lyases I, II, and III. The second set (Table 2) represents an attempt to mimic a sequential-like digestion by dividing each sample into three parts and digesting the first with heparin lyase III, the second with heparin lyases I and III, and the third with heparin lyases I, II, and III. The values in the table are the cumulative percentage of each disaccharide released after each digestion “step.”

**Table 1:** Total disaccharide composition of HS samples after exhaustive digestion.

HS sample	D0H0	D0A0	D0S0	D0A6	D0S6	D2S0	D2S6
Fibroblast	0.8	61.3	18.6	12.0	1.8	3.8	1.7
Epithelial	1.0	59.8	19.2	7.5	1.7	8.2	2.4

Each HS sample was exhaustively digested with heparin lyases I, II, and III at 37°C overnight. Values represent the mole percentage of total disaccharides.

**Table 2:** Cumulative percentage of disaccharides released after sequential-like digestions of HS samples.

HS sample	D0H0	D0A0	D0S0	D0A6	D0S6	D2S0	D2S6
***Fibroblast***							
Heparin lyase III	87.6	79.8	81.4	34.7	31.4	0.0	0.0
Heparin lyases I, III	100.0	80.4	100.0	50.7	100.0	55.3	100.0
Heparin lyases I, II, III	100.0	100.0	100.0	100.0	100.0	100.0	100.0
***Epithelial***							
Heparin lyase III	79.8	66.2	73.2	42.2	20.9	0.0	0.0
Heparin lyases I, III	100.0	71.8	85.6	56.0	86.0	43.3	66.3
Heparin lyases I, II, III	100.0	100.0	100.0	100.0	100.0	100.0	100.0

Each HS sample was divided into three parts. The first was digested with heparin lyase III, the second with heparin lyases I and III, and the third with heparin lyases I, II, and III. Digestions were performed at 37°C overnight. Values represent the cumulative mole percentage of each disaccharide released after each sequential-like digestion step.

### Neutrophil Elastase Inhibition

The ability of various GAG preparations to inhibit the activity of human neutrophil elastase (HNE) was evaluated with an in vitro elastin digestion assay. Inhibitor stock solutions in PBS were made for two commercial products, heparin and heparan sulfate (85 µg/mL), and for two HS preparations from rat pulmonary fibroblasts and epithelial cells (85 µg GAG/mL; determined using the dimethylmethylene blue assay with chondroitin-4-sulfate standards [35]). A quantity (63 µL) of inhibitor stock or PBS was added with vortexing to tubes containing 1 mL of elastin suspension (1.0 mg/mL in PBS), and after 30 minutes at room temperature, a quantity (10 µL) of HNE stock (12.9 µM in PBS) or PBS was added. The resulting set of samples consisted of duplicates of elastin alone, elastin with HNE, and elastin with HNE plus inhibitor at final concentrations of 0.93 mg/mL elastin, 120 nM HNE, and 5.0 µg/mL inhibitor. The tubes were slowly rotated (6 rpm) for 4 hours at 37°C, and the contents were transferred to filter units (Millipore Ultrafree-CL, 0.22 µm) for centrifugation at 3000 *g* for 4 minutes at 4°C. The filtrates (500 µL) were transferred to empty tubes for determination of soluble elastin with the Fastin Elastin assay kit (Biocolor, Carrickfergus, UK). An equal volume of an elastin precipitating reagent was added to each tube with vortexing and allowed to set for 10 minutes. The precipitated elastin was packed by centrifugation (10,000 *g*, 10 min) and drained of liquid. One milliliter of dye reagent containing TPPS (5,10,15,20-tetraphenyl-21,23-porphine tetrasulfonate) was added to each tube with vortexing. The tubes were covered with foil and placed on an orbital mixer (150 rpm) for 90 minutes at room temperature. The insoluble elastin-dye complex was collected by centrifugation (10,000 *g*, 10 min), drained of liquid, and destained with 250 µL of a dye dissociation reagent. The solutions of recovered dye were transferred to a 96-well microplate and read at 513 nm on a SpectraMax 190 microplate reader (Molecular Devices, Sunnyvale, CA). Absorbances of duplicate samples were averaged and corrected by subtracting the elastin control, and elastin content was determined from a standard curve (0–70 µg) based on α-elastin supplied with the kit. The quantity of elastin in the samples was used to calculate values of relative rate (elastin digestion with inhibitor/elastin digestion without inhibitor) for comparing the effect of each GAG preparation on HNE activity.

### Estimation of Hexuronic Acid Epimeric Fractions

The glucuronic acid and iduronic acid components of the unsaturated disaccharides from heparin lyase digestion are listed in Table 3. These epimeric fractions were estimated from published data on the degradation of bovine lung HS [36]. In this reference, disaccharides were measured after three reactions: hydrazinolysis followed by nitrous acid deaminative cleavage at pH 1.5 and 3.9 (reaction 1), nitrous acid deaminative cleavage at pH 1.5 (reaction 2), and exhaustive digestion with a mixture of heparin lyases (reaction 3). In reaction 1, the anhydromannose disaccharides provided glucuronic acid/iduronic acid fractions (*X*_G_, *X*_I_) for all *N*-unsubstituted (H), *N*-acetylated (A), and *N*-sulfated (S) disaccharides of a particular *O*-sulfation in the chain. The anhydromannose disaccharides from reaction 2 gave glucuronic acid/iduronic acid fractions (*x*_GS_, *x*_IS_) for *N*-sulfated disaccharides of a particular *O*-sulfation in *N*-sulfated blocks, and this was assumed representative of the overall chain. The unsaturated disaccharides from reaction 3 were a source of fractions for *N*-unsubstituted, *N*-acetylated, and *N*-sulfated disaccharides (*Y*_H_, *Y*_A_, *Y*_S_) of a particular *O*-sulfation in the chain. With this information, it was possible to write a mass balance on disaccharides with the same degree of *O*-sulfation for either glucuronic acid
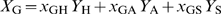
(1)or iduronic acid
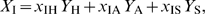
(2)where *x*_GH_ and *x*_IH_ are fractions of glucuronic acid and iduronic acid, respectively, for *N*-unsubstituted disaccharides of a particular *O*-sulfation, and *x*_GA_ and *x*_IA_ are fractions of glucuronic acid and iduronic acid, respectively, for *N*-acetylated disaccharides of a particular *O*-sulfation.

**Table 3:** Estimated fractions of glucuronic acid and iduronic acid in unsaturated HS disaccharides.

Hexuronic acid	D0H0	D0A0	D0S0	D0A6	D0S6	D2S0	D2S6
Glucuronic	1.00	0.99	0.83	0.54	0.61	0.01	0.00
Iduronic	0.00	0.01	0.17	0.46	0.39	0.99	1.00

Epimeric fractions were estimated from experimental data on bovine lung HS [36] as described in Materials and Methods. Values represent the mole fraction of each disaccharide that is glucuronic acid or iduronic acid.

In the study on bovine lung HS [36], *N*-unsubstituted glucosamine was not detected in the analysis of the unsaturated disaccharides from reaction 3. This information (*Y*_H_ = 0) was used with the other data in the report to solve Equations 1 and 2 for the glucuronic acid/iduronic acid components of disaccharide groups containing *N*-acetylated glucosamine (D0A0 and D0A6). For D0A0, a mass balance was written on all non-*O*-sulfated disaccharides, and for D0A6, a mass balance was written on all 6-*O*-sulfated disaccharides. The determination of the glucuronic acid/iduronic acid components for disaccharide groups with *N*-sulfated glucosamine (D0S0, D0S6, D2S0, and D2S6) was straightforward, using the reported data from reaction 2 [36]. The remaining disaccharide group containing *N*-unsubstituted glucosamine (D0H0) was assumed to be all glucuronic acid.

### Heparin Lyase Specificities

**Figure 1:**
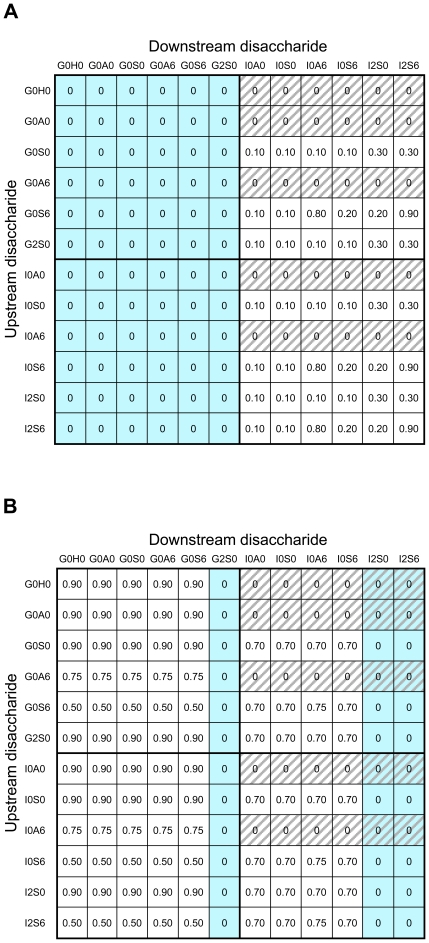
Heparin lyase cleavage matrices for HS disaccharide-disaccharide linkages. (**A**) Estimated cleavage probabilities for heparin lyase I. (**B**) Estimated cleavage probabilities for heparin lyase III. Each cell in (**A**) and (**B**) represents a disaccharide-disaccharide linkage with an indicated probability of cleavage by the specific heparin lyase. Turquoise cell  =  linkage not cleaved by lyase. Crosshatched cell  =  linkage prohibited by rules of biosynthesis.

The 12×12 cleavage matrices for heparin lyases I and III are shown in Figure 1. For each matrix, the rows (*i*) and columns (*j*) are assigned to the 12 disaccharides identified by Table 3, and each cell (*i, j*) represents a linkage between disaccharides *i* and *j*, with *j* being the downstream disaccharide (closer to the tetrasaccharide-protein linker). Some linkages (crosshatched cells) are prohibited by the rules of biosynthesis (rows 1, 2, 4, 7, and 9 in the right half of the matrix), and other linkages (turquoise cells) are outside the rules of lyase specificity. The entry in each cell indicates the probability of cleavage by the lyase of interest. Wherever possible, these values were adapted from reported data on the fractional conversion of substrates containing the linkages [37], [38]. Values of complete conversion were adjusted to 0.9 to allow for some randomness in the simulated cleavage process. When conflicting information was found in the literature, the most inclusive rules for lyase specificity were selected [30], [39]–[42].

The cleavage matrix for heparin lyase I in Figure 1A reflects a specificity for iduronic acid in the downstream disaccharide [37], [40]. Although evidence suggests that cleavage also occurs with G2S6 on the downstream side [42], this disaccharide was absent from the HS composition (Table 3). Accordingly, all entries in the left half of the matrix (columns 1–6) were set to zero. Five linkages (S0-I2, S6-I0A6, S6-I0S6, S6-I2S0, and S6-I2S6) were assigned cleavage probabilities from 0.2 to 0.9 based on reported degradation of defined oligosaccharides [38]. Other linkages containing unsulfated iduronic acid (S0-I0, S6-I0A0, and S6-I0S0) were given a nominal probability of 0.1.

Figure 1B shows the cleavage matrix for heparin lyase III. The specificity of this lyase requires a downstream disaccharide containing either unsulfated glucuronic acid (primary site) or unsulfated iduronic acid (secondary site) [37], [38], [42]. As a result of this requirement, the entries in columns 6, 11, and 12 of the cleavage matrix were set to zero. Cleavage probabilities for other cells were established from experimental degradation results on defined oligosaccharides containing four linkages (S0-G0, A6-G0, S6-G0, and S6-I0) [38]. These probabilities ranged in value from 0.5 to 0.9. For several linkages (H0-G0, A0-G0, and S0-I0), comparable data were not found, and probabilities were estimated at values from this range.

The cleavage matrix for heparin lyase II is not shown because all the entries were adjusted to a value of 1. As the least discriminating of the lyases [37], this enzyme was restricted to the last position in the simulated digestion sequence. Consequently, the lyase was assumed to finish the degradation of the HS chain and was allowed to break every intact disaccharide-disaccharide bond. This assumption seemed reasonable since exhaustive digestion with all three lyases is a common preparatory procedure for determining the disaccharide composition of HS oligosaccharides. However, if heparin lyase II were applied at an earlier step in the sequential digestion, the cleavage matrix would require some modification to more accurately reflect the action of this lyase.

### Computational Analysis of HS

The computational approach for predicting patterns of HS domain organization was driven by experimental inputs and user-defined rules of HS biosynthesis and lyase breakdown. In its present form, the model uses the total disaccharide composition determined after complete lyase digestion and the relative amounts of various disaccharides released after individual or sequential lyase digestion in conjunction with average chain length measurements to build populations of theoretical HS chains to represent the original HS sample. These HS chain populations are then evaluated for structural patterns of domain organization. The model performs these operations through a modular format consisting of input/output sections and three routines called chainmaker, chainbreaker, and chainsorter.

#### Input

The input section of the computational model requires information on (a) the number of disaccharides in the chain, *N*, (b) the number of chains, *M*, (c) the total disaccharide composition with glucuronic acid/iduronic acid specifications, (d) the order of heparin lyase digestion, (e) the breakdown constraints for matching simulated and real results of disaccharide release after each digestion step, and (f) the specific disaccharides that define each domain.

#### Chainmaker routine

**Figure 2:**
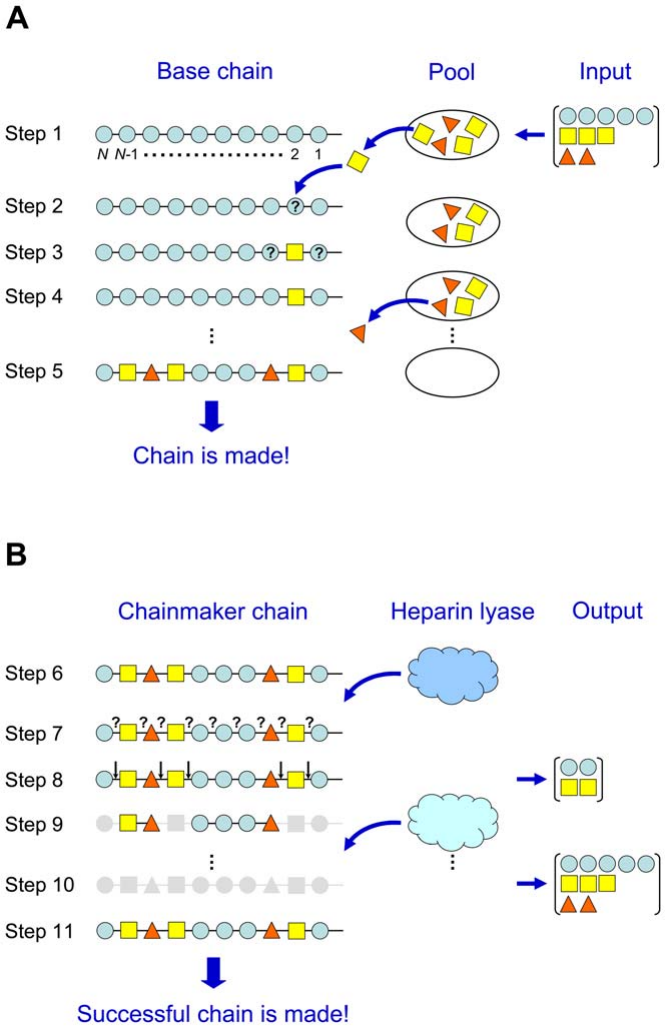
Schematic diagrams of chainmaker and chainbreaker routines for generating HS chains. (**A**) Chainmaker routine: (1) creates a base chain of *N* units of G0A0; (2) selects a disaccharide randomly from a pool based on experimental composition and places the disaccharide at a random position on the chain; (3) checks the positional constraints for disaccharide placement; (4) repeats steps 2–3 until placement occurs; (5) repeats steps 2–4 until all disaccharides from the pool are placed on the chain. The completed chain moves to the chainbreaker routine. (**B**) Chainbreaker routine: (6) receives the completed chain from the chainmaker routine; (7) initiates the first digestion (heparin lyase I or III) by comparing a random probability with the cleavage probability of each bond; (8) determines the broken bonds and releases disaccharides with cleavage on both sides; (9) repeats steps 7–8 for the second digestion; (10) cleaves all remaining bonds and releases all remaining disaccharides in the final digestion (heparin lyase II); (11) compares the released disaccharides from the first two digestions with the experimental breakdown constraints. If the constraints are satisfied, the successful chain moves to storage.

The chainmaker routine (Figure 2A) creates an HS chain of specified length and composition with disaccharides situated in positions that satisfy known rules of biosynthesis. In step 1 of the routine, a base chain is generated containing *N* units of unmodified disaccharide (G0A0) (see definition of nomenclature in Materials and Methods). The actual disaccharides in the chain are calculated from the total disaccharide composition, and these disaccharides (except for G0A0) are placed into a selection pool. A disaccharide is randomly selected from the pool, and a position on the chain is randomly chosen (step 2). If G0A0 exists in the selected chain position and *N*-unsubstituted glucosamine is not on the upstream side (farther from the tetrasaccharide-protein linkage), the selected disaccharide replaces G0A0. If *N*-unsubstituted glucosamine is on the upstream side of the position, the selected disaccharide must contain glucuronic acid in order to replace G0A0. If any of these conditions is not satisfied, another position is randomly chosen for the disaccharide until a successful replacement occurs.

Once the selected disaccharide is placed on the chain, it is subjected to another set of positional constraints (step 3). These constraints are adapted from reported observations on HS biosynthesis [8], [36], [37], [43] and consist of the following: (a) a disaccharide with either iduronic acid or 2-*O*-sulfation must have *N*-sulfated (±6*S*) glucosamine on the upstream side, (b) a disaccharide with 6-*O*-sulfation must have at least one adjacent disaccharide with *N*-sulfated (±6*S*) glucosamine, and (c) a disaccharide with *N*-unsubstituted glucosamine must have glucuronic acid on the downstream side. If the positional constraints are met, the disaccharide remains on the chain (step 4); otherwise, the disaccharide returns to the pool, G0A0 refills the vacated spot, and the selection process starts over. Eventually, all disaccharides from the pool are placed on the chain, and the resulting chain has a disaccharide composition compatible with the original input (step 5).

#### Chainbreaker routine

The chainbreaker routine (Figure 2B) determines whether a newly created chain (step 6) is acceptable by subjecting the chain to simulated digestions and comparing the released disaccharides with breakdown constraints. The chain is exposed to sequential digestion by heparin lyases in a specified order (III, I, II or I, III, II). In the first digestion step (step 7), the linkage between the first and second disaccharides on the chain is assigned a probability of cleavage based on the specificity of the particular lyase [30], [37]–[42] (Figure 1 and Materials and Methods). A random number is generated, and if it is less than or equal to the assigned probability, the bond is broken. This process is repeated along the chain, assessing each disaccharide-disaccharide linkage, until the end is reached. The chain is re-examined for all broken bonds (step 8), and if cleavage occurs on both sides of a disaccharide, that disaccharide is considered “released.” The second digestion step is then activated (step 9), and the chain is reprocessed with a new set of cleavage probabilities appropriate for the lyase of the step. Finally, in the last digestion step (step 10), all bonds remaining in the chain are broken.

The disaccharides released in each of the first two digestion steps are compared with the experimental products of sequential digestion, and if they fall within the allowable limits (breakdown constraints), the chain is designated as a successful chain (step 11). If any constraint is not met, the chain is discarded, and the program returns to the chainmaker routine to generate a new chain. The program cycles between chainmaker and chainbreaker routines until *M* successful chains are generated.

#### Chainsorter routine

The chainsorter routine transforms each successful chain into a string of user-defined domains. Each chain is processed disaccharide by disaccharide, classifying the disaccharide into the appropriate domain and keeping track of the length and order of the contiguous stretches of each domain along the chain.

#### Output

The output section of the model generates files that contain the following: (a) a list of successful chains in the form of sequences of numbers, each number representing a particular disaccharide, (b) a list for each domain giving a sequence of numbers for each chain, where each number indicates the domain length in disaccharides sequentially along the chain, and (c) a summary containing the successful chains, the disaccharide breakdown of these chains after specific lyase digestion, and the average domain lengths within these chains.

### Computer Program

A computer program of the model was written in Fortran 95 using Absoft Pro Fortran software (Absoft, Rochester Hills, MI) and supported by the IMSL Fortran Library (Visual Numerics, Houston, TX). Pseudorandom uniform numbers were generated using a generalized feedback shift register to avoid cycling issues. This generator was essential for building unique chains; for example, approximately one million chains were required in order to produce 100 successful chains (no duplicates) of 250 disaccharides for fibroblast HS. The program was compiled and executed on a personal computer operating under Windows XP (Microsoft, Redmond, WA) with an Intel Core 2 Quad processor (Intel, Santa Clara, CA) at 2.40 GHz (3.50 GB of RAM). Execution times varied from less than one minute to more than two days, depending on the values for chain length, chain number, and breakdown constraints.

### Domain Analysis

The average domain size in disaccharides for chains of equal length was calculated by determining the average domain size for each chain and then averaging these values over all the chains in the set. The 95% confidence interval for the average domain size was estimated from the standard error of the mean and the Student's *t*-distribution. The characteristics of the plateau region (equilibrium domain size; critical length) were determined by a one-way analysis of variance (alpha  = 0.05) of the points in each plot. If there was a statistical difference (*p*<0.001), a pairwise multiple comparison using the Holm-Sidak method (overall significance level  = 0.05) was performed. The points that showed no statistical differences among themselves were averaged to obtain the equilibrium domain size, and the shortest chain length in the group was designated as the critical length for stabilization of the domain. To determine if there were statistical differences (*p*<0.001) between the domains of the two sets of HS chains, a *t*-test was performed (alpha  = 0.05) on the less sulfated domains and on the highly sulfated domains.

### Fourier Analysis

Fourier analysis was completed on a set of *M* chains of length *N* disaccharides, where *N* is a power of 2. The disaccharide sequence of each chain was converted into a binary sequence representing the domains of interest [44], [45]. A fast Fourier transform routine in Microsoft Office Excel 2003 was applied to each binary sequence to yield a set of *N* complex coefficients
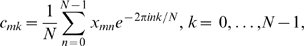
(3)where *k* is the wavenumber, *k*/*N* is the spatial frequency, and *x_mn_* is a component in the binary sequence *m* (*m* = 1, …, *M*) at position *n* (*n* = 0, …, *N*-1). For frequencies above the base frequency (*k* = 0), the power spectrum for each sequence was calculated as the square of the magnitude of the Fourier coefficients
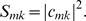
(4)


An average power spectrum for a set of *M* spectra [46] was defined as a frequency by frequency average over all spectra
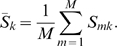
(5)


For each HS sample, 100 chains were generated having a length equal to the smallest power of 2 above the critical length. Each chain was converted into a binary sequence by substituting 1 for disaccharides in the highly sulfated domain and 0 for disaccharides in the less sulfated domain. The Fourier coefficients (Equation 3) and the resulting power spectrum (Equation 4) were calculated for each binary sequence, and the power spectra were averaged (Equation 5), starting with 10 chains and increasing in 10-chain increments to 100 chains, to determine a set of average power spectra for each HS sample. Each average power spectrum was normalized with respect to the highest response and plotted to show only the first half of the symmetric trace (*k* = 1, …, *N*/2). A prominent peak at a wavenumber of *k* signifies a periodic structure in the chain consisting of *N*/*k* disaccharides. The power at *k* = 0 contains no information on position and was disregarded for this analysis.

## Results

### Computational Analysis of HS Preparations

HS chains from rat pulmonary fibroblasts and epithelial cells [34] were subjected to chemical compositional analysis, and the data were used to generate predicted chain structures using the computational model. The experimental data consisted of the total disaccharide composition after digestion with heparin lyases I, II, and III (Table 1) and the percentage of each disaccharide released after sequential-like digestions with heparin lyases III, I, and II (Table 2). Epimeric fractions (Table 3) were estimated from published data [36] and used to proportion the unsaturated disaccharides in Table 1 into glucuronic acid and iduronic acid components. Three groups (D0A0, D0A6, and D2S0) in Table 2 were chosen for comparison with the simulated disaccharide release, and practical tolerances for the breakdown constraints were set at D0A0 ±2%–4%, D0A6 ±20%, and D2S0 ±0% for each of the first two digestion steps. The resultant output from the model was a series of chains of a given length and disaccharide sequence that were consistent with the experimental data (Tables 1 and 2). The chains were analyzed in several ways to predict general patterns of domain organization as well as the distribution and density of specific structural motifs within the two HS populations.

### Predictions of General Patterns

**Figure 3:**
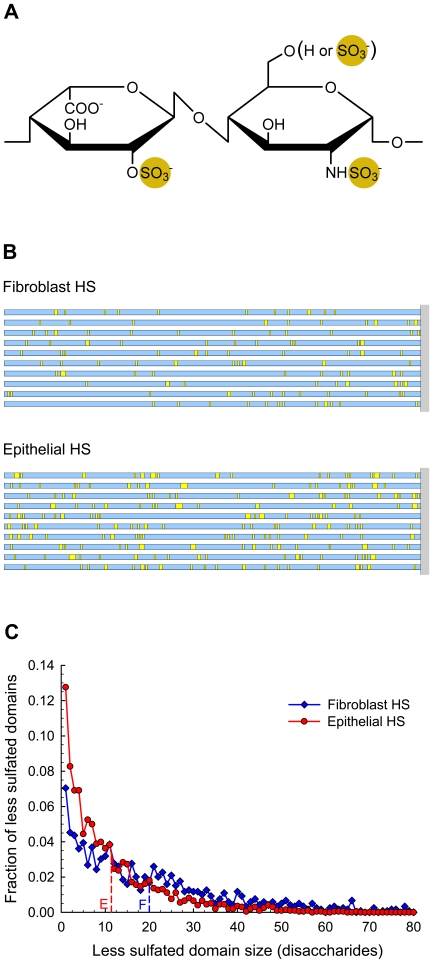
General patterns of domain organization predicted for fibroblast HS and epithelial HS. (**A**) Chemical structure of the disaccharide containing 2-*O*-sulfated iduronic acid that defines the highly sulfated domain. All other disaccharides, including the remaining sulfated disaccharides, belong to the less sulfated domain. (**B**) Schematic diagram of HS chains showing domain organization. Yellow blocks are highly sulfated domains; blue blocks are less sulfated domains. Chain lengths are 250 disaccharides. Minimum block length is 1 disaccharide. (**C**) Size distribution of less sulfated domains in HS chains. *N* = 250 disaccharides and *M* = 100 chains. Although the distribution is shown for less sulfated domains up to 80 disaccharides in length, a few longer domains are present in both sets of HS chains. For epithelial HS, less sulfated domains extend to 87 disaccharides; for fibroblast HS, less sulfated domains extend to 113 disaccharides. Dashed lines indicate average sizes: E = 11.4±0.2 disaccharides (average ±95% confidence limits) for epithelial chains and F = 20.0±0.4 disaccharides for fibroblast chains.

HS structure has been described as being comprised of alternating domains of highly sulfated regions (S-domains) separated by less sulfated regions (NA-domains) which are generally connected by regions containing intermediate levels of sulfation (transition domains). However, HS domains can be arbitrarily specified to include any defined structures of particular disaccharides. Thus, as a first approximation for this analysis, domains were defined simply as being one of two regions—a highly sulfated domain containing disaccharides with 2-*O*-sulfated iduronic acid (Figure 3A) and a less sulfated domain containing all other disaccharides. Consequently, the less sulfated domain contains not only unsulfated disaccharides but also some sulfated disaccharides (e.g., I0S0, I0S6, and G0A6). In terms of commonly used nomenclature, the highly sulfated domain is most similar to the S-domain, and the less sulfated domain encompasses both the NA-domain and the transition regions [30].

Examples of 250-disaccharide chains with alternating regions of highly sulfated domains (yellow blocks) and less sulfated domains (blue blocks) are illustrated for both fibroblast and epithelial HS chains (Figure 3B). The less sulfated domains exhibited a wide range of sizes (1–113 disaccharides) while the highly sulfated domains were more limited in size (1–4 disaccharides). Comparison of the size distributions of the less sulfated domains (Figure 3C) for 100 chains of each sample shows a larger fraction of shorter domains (below 10 disaccharides) for epithelial HS chains and a larger fraction of longer domains (above 20 disaccharides) for fibroblast HS chains. For a more in-depth analysis of the overall patterns of domain organization that characterize these chains, two analytical methods were applied to the output. The first method was a relatively straightforward technique using average domain sizes, and the second method was a more sophisticated technique of pattern recognition using Fourier analysis.

#### Average domain sizes

**Figure 4:**
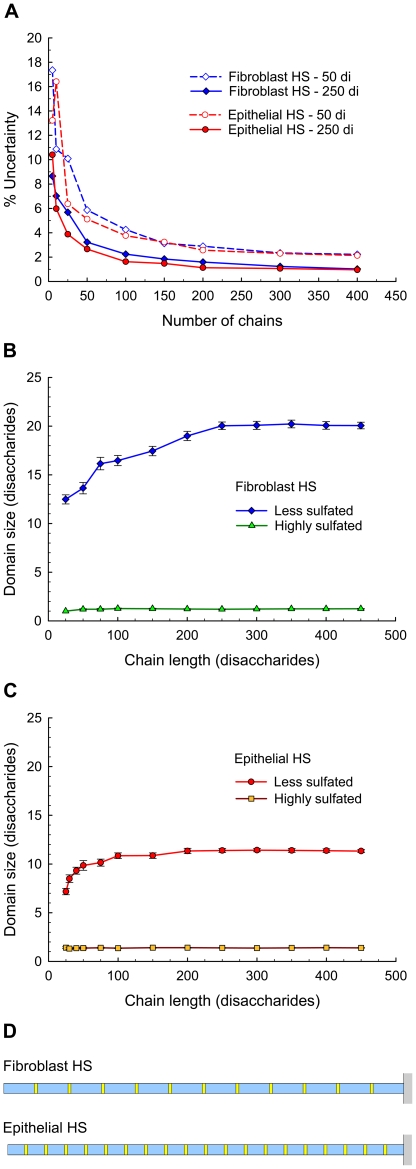
Average domain sizes predicted for fibroblast HS and epithelial HS chains. (**A**) Uncertainty in average domain size of less sulfated domains as a function of chain number. *N* = 50 and 250 disaccharides; *M* = 5–400 chains. % Uncertainty  =  [(±95% confidence limits)/(average domain size)] ×100. (**B**) Average domain size as a function of chain length for fibroblast HS. (**C**) Average domain size as a function of chain length for epithelial HS. *N* = 25–450 disaccharides and *M* = 100 chains for (**B**) and (**C**). Error bars show 95% confidence interval. (**D**) Schematic diagram of chains showing predicted domain patterns. Yellow blocks are highly sulfated domains; blue blocks are less sulfated domains. Each chain is above the critical length, and domain sizes are equilibrium values. For fibroblast HS, less sulfated domain  = 20.1±0.1 disaccharides (average ±95% confidence limits) and highly sulfated domain  = 1.22±0.02 disaccharides. For epithelial HS, less sulfated domain  = 11.2±0.2 disaccharides and highly sulfated domain  = 1.38±0.02 disaccharides.

The simple determination of average domain sizes requires a sufficient number of chains to achieve a reasonable level of consistency in the predicted results. A series of runs was completed in which 5 to 400 chains of various lengths were generated for both fibroblast and epithelial samples. The results for the shortest chains (50 disaccharides) and the longest chains (250 disaccharides) are shown in Figure 4A, where the uncertainty in the average size of the less sulfated domain is plotted as a function of the number of generated chains. Although the uncertainty was as high as 18% for 10 or less chains, the generation of at least 100 chains reduced the uncertainty to less than 5%. Further increases in the number of chains decreased the uncertainty even more, but this gain was offset by growing computational time. Based on these results, the generation of 100 chains guaranteed a reasonable run-to-run consistency, and all subsequent executions were carried out with this number of chains.

Analysis of the average size of the less sulfated domain as a function of chain length (Figures 4B and 4C) revealed the same overall trend for both samples—domain size increased with increasing chain length until a critical length was reached, after which the domain size remained relatively constant. For fibroblast HS chains (Figure 4B), this critical chain length was 250 disaccharides, and the equilibrium domain size was 20.1±0.1 disaccharides (average ±95% confidence limits). For epithelial HS chains (Figure 4C), the critical chain length occurred at a much shorter 100 disaccharides, and the equilibrium domain was much smaller at 11.2±0.2 disaccharides. In contrast, the size of the highly sulfated domain remained relatively stable regardless of chain length. The equilibrium size was 1.22±0.02 and 1.38±0.02 disaccharides for fibroblast and epithelial HS chains, respectively (Figures 4B and 4C). The equilibrium sizes of both the less sulfated domains and the highly sulfated domains were significantly different (*p*<0.001) between the two HS samples. A comparison of the resulting patterns of these domains in a fibroblast HS chain and an epithelial HS chain above the critical length reveals that the epithelial HS chain has a greater frequency of highly sulfated domains separated by shorter segments of less sulfated domains (Figure 4D).

#### Fourier analysis

**Figure 5:**
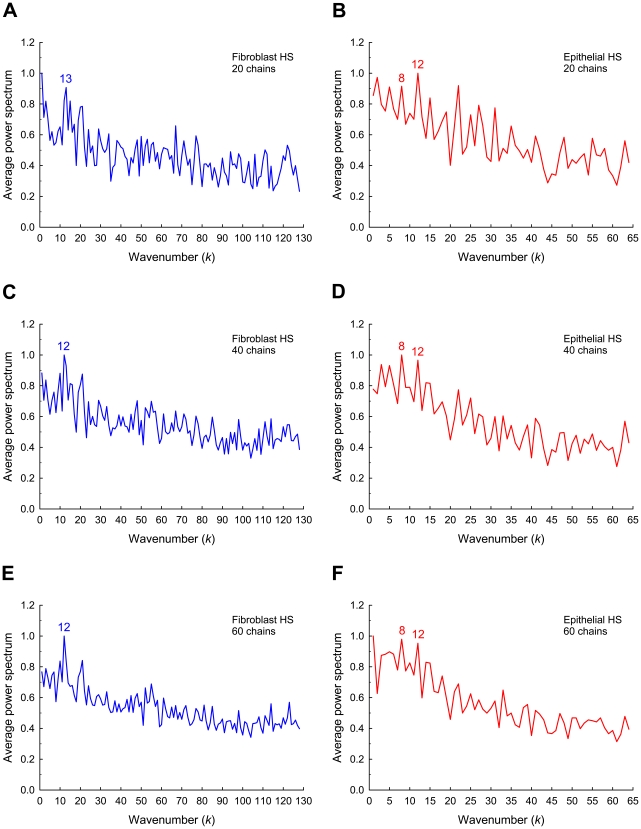
Examples of average Fourier power spectra for HS chains. Average power spectrum for fibroblast HS based on (**A**) 20 chains, (**C**) 40 chains, and (**E**) 60 chains. *N* = 256 disaccharides. Average power spectrum for epithelial HS based on (**B**) 20 chains, (**D**) 40 chains, and (**F**) 60 chains. *N* = 128 disaccharides. Each spectrum is normalized with respect to the highest response and is shown for the first half of the symmetric trace (*k* = 1, …, *N*/2).

Because discrete Fourier transform analysis has been successfully used to detect periodical properties in DNA and protein sequences [45], [46], this technique was examined as an alternative method for discerning general patterns of domain organization in HS chains. For each HS sample, 100 chains were generated, and the average Fourier power spectrum was calculated for a variable number of chains, starting at 10 and increasing by increments to 100. A few examples of the resulting power spectra for each group of HS chains are depicted in Figure 5.

The average power spectra for fibroblast HS chains of 256 disaccharides consistently showed a dominant peak at a wavenumber of *k* = 12 or 13 (Figures 5A, 5C, and 5E). This peak was visible in all the average spectra from as few as 10 chains to as many as 100 chains. The peak wavenumbers indicate a periodic component (*N*/*k*) with a length of 20–21 disaccharides. This size is in good agreement with the equilibrium domain sizes (Figure 4B), where the length of one highly sulfated domain followed by one less sulfated domain is 21 disaccharides.

The patterns of the average power spectra for epithelial HS chains of 128 disaccharides were more complex to interpret (Figures 5B, 5D, and 5F). The average spectra showed two peaks at *k* = 8 and 12 that were predominant in the majority of the spectra. These wavenumbers represent periodicities of 16 and 11 disaccharides, suggesting a mixture of two patterns. Remarkably, the average of these two numbers compares favorably with the periodic spacing of 13 disaccharides predicted from the equilibrium domain sizes (Figure 4C).

### Predictions of Specific Motifs

Some activities of HS are likely related to the general organization of the chains, while others are dependent on the presence of specific local structural motifs. As an extreme example, the ability of heparin to bind to antithrombin III is dependent on the presence of a rare pentasaccharide structure [10], whereas most other protein interactions seem to involve less stringent criteria [3], [5], [9], [12]. The ability of the computational model to predict the arrangement of particular structural motifs was evaluated by analyzing the HS chains for the presence of structures associated with elastase inhibition.

Heparin is a potent inhibitor of neutrophil elastase [47]–[50]. In particular, the inhibition of elastase-mediated solubilization of elastin has been shown to depend on both the presence of sulfated disaccharides and the length of the chain [48]. The removal of 2-*O*-sulfate from the hexuronic acid residues in heparin reduced inhibitory activity by 20%, while the removal of 6-*O*-sulfate or *N*-sulfate from the glucosamine residues resulted in approximately 50% and 70% loss of inhibitory activity, respectively [48]. Examination of the molecular structure of heparin [26] revealed that these three sulfate groups form a cluster that alternates from side-to-side along the length of the chain. The sulfate cluster is composed of two sequential disaccharides on the chain—a disaccharide with 2-*O*- and 6-*O*-sulfation followed by an upstream disaccharide with *N*-sulfation. Molecular-docking simulations suggested that these sulfate clusters bind to positively charged, clamp-like regions on elastase that span the active site [48]. According to this bridging model, appropriately sized heparin chains would tend to block the active site and cause inhibition, whereas shorter chains would be unable to span the site. Longer chains would provide increased stability in blocking the active site and might eventually encourage translation of the elastase molecule along the chain, thus contributing to an increased inhibitory response with increased chain length. This proposed mechanism was supported by experimental results with heparin oligosaccharides. No significant effect on elastolysis was detected until the chain reached a length of about 6 disaccharides (16% inhibition), and this effect increased by more than three times (51% inhibition) with the addition of one disaccharide to the chain. Although longer oligosaccharides (greater than 7 disaccharides) were not tested, inhibitory activity leveled off at about 92%–95% with heparin preparations having average chain lengths of 24–30 disaccharides [48].

**Figure 6:**
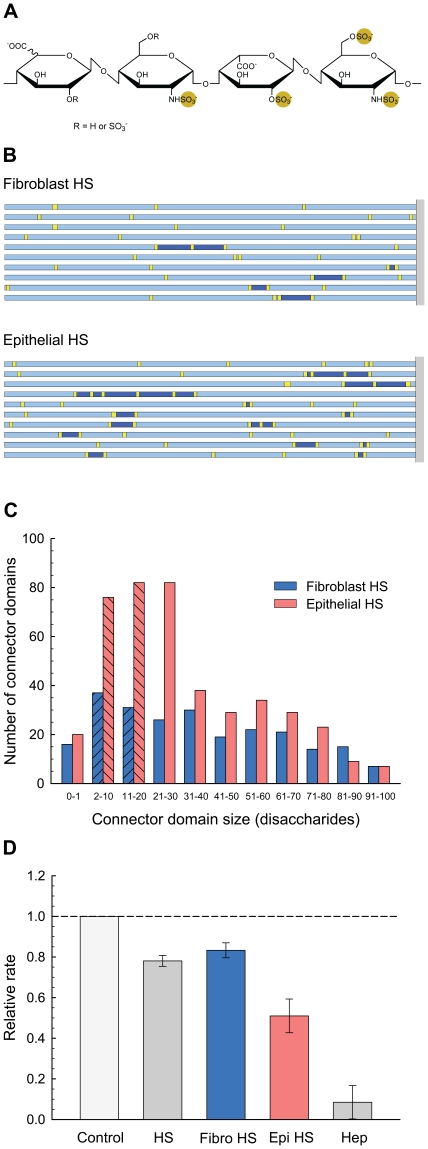
Specific motif for inhibition of elastolysis predicted for fibroblast HS and epithelial HS. (**A**) Chemical structure of the disaccharide pair that defines the cluster domain. Disaccharides excluded from the cluster domain belong to the connector domain. (**B**) Schematic diagram of HS chains showing domain organization with specific motif for inhibition of elastolysis. Yellow blocks are cluster domains; blue blocks are connector domains; dark blue blocks are connector domains that meet the size requirement for effective elastase inhibition. Chain lengths are 250 disaccharides. Minimum block length is 1 disaccharide. (**C**) Size distribution of connector domains in HS chains. *N* = 250 disaccharides and *M* = 100 chains. Crosshatched bars indicate lengths of connector domains (2–20 disaccharides) for effective elastase inhibition. Although the distribution is shown for connector domains up to 100 disaccharides in length, longer domains are present in both sets of HS chains. For fibroblast HS, connector domains extend to 226 disaccharides with an average size of 52±5 disaccharides (average ±95% confidence limits). For epithelial HS, connector domains extend to 184 disaccharides with an average size of 36±3 disaccharides. (**D**) Inhibition of elastolysis by GAG preparations. Relative rate  = (elastin digestion with inhibitor)/(elastin digestion without inhibitor). Bar height equals the average of duplicate readings; error bar shows the propagation-of-error estimate using standard errors. Control  =  no inhibitor; Hep  =  commercial heparin (17–19 kDa); HS  =  commercial heparan sulfate (8–10 kDa); Fibro HS  =  HS preparation from rat pulmonary fibroblasts; Epi HS  =  HS preparation from rat pulmonary epithelial cells. Reaction conditions: [Inhibitor]  = 5.0 µg/mL; [HNE (human neutrophil elastase)]  = 120 nM; [Elastin]  = 0.93 mg/mL; buffer  =  Dulbecco's phosphate-buffered saline without calcium and magnesium salts; temperature  = 37°C; time  = 4 hours; volume  = 1.073 mL.

On the basis of this information, search criteria were specified for elastase inhibitory motifs in HS chains. A pattern was defined as a sulfate cluster (cluster domain) containing a pair of disaccharides, with I2S6 as the downstream disaccharide and *N*-sulfated disaccharide as the upstream disaccharide (Figure 6A), separated by 2–20 disaccharides (connector domain) from the next sulfate cluster. Cluster/connector/cluster motifs having connector domains larger or smaller than this range were considered ineffective for elastase inhibition. Even though there was no experimental evidence in support of this size constraint for effective elastase inhibitory motifs in HS chains, the existence of limits seemed reasonable based on structural considerations. For example, a connector domain of less than 2 disaccharides would create a cluster/connector/cluster length of less than 6 disaccharides. A chain section with this specific motif would have difficulty in physically spanning the active site of elastase as suggested by the bridging model for heparin inhibition [48]. Longer connector domains would allow this bridging to take place with subsequent disruption of elastase activity. However, at some point, unlike the heparin chain, the connector domain would become too long, leading to reduced effectiveness of the HS chain. The proposed divergence from the trend observed with heparin oligosaccharides was attributed to the alternating domain structure of the HS chain. In contrast to the relatively rigid heparin chain, the HS chain would have increased flexibility because of the less sulfated and therefore less constrained connector domains [1], [26], [27]. For an HS chain in the bridging formation, a flexible connector domain would have less likelihood of remaining in close proximity to the elastase surface. This situation would allow substrate access to the active site, a condition that would become worse with increasing size of the connector domain. In fact, a long connector domain might even prevent the cluster domains from binding to the requisite elastase regions for bridging the active site. The upper limit of the effective connector domain was set to a relatively high value of 20 disaccharides as a first step in the analysis. The resulting cluster/connector/cluster motif of 24 disaccharides agreed with the average chain size of an experimentally evaluated heparin preparation that was an effective elastase inhibitor [48].

Sequences from the 250-disaccharide chains generated for Figure 3B were searched for the distinctive cluster/connector/cluster pattern of the elastase inhibitory motif. Examples of 10 chains from each HS sample are illustrated in Figure 6B, showing cluster domains (yellow blocks), connector domains (blue blocks), and connector domains that meet the size requirement of the pattern (dark blue blocks). Comparison of the two sets of chains clearly shows that epithelial HS contained more chains with the elastase inhibitory motif and more instances of this motif per chain than fibroblast HS. The statistics for 100 chains confirmed these findings, indicating that 89% of epithelial chains and 54% of fibroblast chains had the motif, and within those selected chains, 58% of epithelial chains and 24% of fibroblast chains had more than one occurrence of the motif. Figure 6C shows the size distribution of connector domains in the 100 chains generated for each HS sample. Although the plots are shown for connector domains up to 100 disaccharides in length, the actual distributions extend past this cut-off for both sets of chains. The average connector domain size (average ±95% confidence limits) was 36±3 disaccharides for epithelial HS and 52±5 disaccharides for fibroblast HS. The crosshatched bars represent the connector domains that fit the requirements for the elastase inhibitory motif. They indicate that epithelial HS had more than twice as many of these motifs as fibroblast HS. This same trend was observed in the subset of domains with connector lengths of 2–10 disaccharides. Although the original limits on the connector domain were generously set at 2–20 disaccharides, the lower end (2–10 disaccharides) is more likely to be the functional range for inhibition based on elastase structural considerations.

### Elastase Inhibition by HS Preparations

Based on the relative density of the elastase inhibitory motif, the model results predict that epithelial HS would be a more potent inhibitor of elastase than fibroblast HS. This prediction was tested by measuring elastase activity in the presence of various GAG preparations consisting of commercial heparin and heparan sulfate and HS preparations derived from rat pulmonary fibroblasts and epithelial cells. The results from the experiments are presented in Figure 6D in terms of the relative rate of reaction (elastin digestion with inhibitor/elastin digestion without inhibitor). As expected, heparin was a potent inhibitor of elastolysis (92% inhibition) while heparan sulfate was less active (22% inhibition). Interestingly, the HS preparations from the pulmonary cells showed differences consistent with the model predictions. Whereas the HS preparation from the fibroblasts reduced elastase activity by only 17%, the epithelial HS preparation was significantly more effective at 49% inhibition.

## Discussion

Glycans are a diverse group of carbohydrates that play an intricate role in fundamental physiological processes through their modulation of protein activity and their ability to fine-tune biological responses [4]. Sulfated glycosaminoglycans represent a group of linear sugars that are incorporated as proteoglycans at the surfaces of cells and in the extracellular matrix. GAGs positioned at the cell-extracellular matrix interface have a unique opportunity to interact with a wide spectrum of proteins, including growth factors, cytokines, chemokines, morphogens, proteases, antiproteases, cell adhesion molecules, and extracellular matrix components [1], [8]. The resulting GAG-protein interactions provide a mechanism by which GAGs exert their control over critical biological processes. However, unlike the binary on/off concept generally applied to the understanding of protein activity regulation (e.g., receptor binding and activation), GAGs exhibit a gradation of control through the diverse nature of their disaccharide sequence, chemical organization, and chain length. The inherent heterogeneity of GAG chains is a product of nontemplate-based biosynthesis [5]. The ability to characterize this complexity remains a challenge with the current analytical methods, and as a consequence, progress in deciphering GAG structure-function relationships has been hindered.

The most structurally complex member of the GAG family is heparan sulfate, having an information-dense chain carrying potentially 48 different disaccharide structures segregated into chemical blocks of highly sulfated and largely unsulfated domains [1], [8], [9]. Heparan sulfate is a major physiological player at the cell-extracellular matrix interface where it has been shown to interact with and mediate the activity of a number of proteins. Many HS-protein interactions appear to be less dependent on the specific disaccharide sequence and more dependent on the domain organization of the HS chain [3], [5], [9], [12]. Because the analytical capability to detect domain organization is currently limited, new methods that can provide insight into HS chain organization will be extremely useful to researchers in the field.

Several early attempts with mathematical modeling [51] and computer simulation [52]–[55] were focused on the structure of heparin. These methods were used in conjunction with cleavage experiments to test alternative hypotheses concerning the action pattern of heparin lyase I and the arrangement of specific oligosaccharides within the heparin chain. Although the methodology was later applied to hyaluronic acid and the action of hyaluronate lyase [56], the extension of these techniques to the more complex structure of HS was not implemented.

The computational approach described in this study offers a unique way to probe the organizational structure of HS chains. Using minimal experimental data from disaccharide analysis and selective heparin lyase digestion, the computational routines can generate chains according to rules of HS biosynthesis and lyase specificity and then transform them into strings of user-defined domains for pattern analysis. As demonstrated with HS chain populations from two different cell culture sources, the model has the ability to predict significant differences in overall domain organization properties as well as in the density and distribution of specific functional motifs. HS activity measurements revealed that these structural differences are related to functional differences in HS-protein interactions. Hence these tools can be used in conjunction with experimental measurement to investigate the relationship between proposed structural requirements and functional activities in HS.

The existence of a causal relationship between cell-surface HS structure and cell behavior is supported by various studies. HS chains from different cell types have been reported to have consistent structural variations that result in distinct biological functions [57]–[59]. For example, experimental measurements of HS chains purified from the surfaces of mouse mammary gland epithelial cells and embryonic fibroblasts showed differences in structure (chain length and disaccharide composition of highly sulfated domains) as well as binding to type I collagen [58]. The current investigation is consistent with these earlier studies and provides additional evidence for cell type-specific differences in HS structure that have direct functional consequences. In this case, however, these differences include not only analytical measurements of disaccharide compositions for the chain and lyase-specific domains but also computer-predicted patterns of overall domain structure and specific functional motifs. On both a large and small scale, the differences in HS structure appear to contribute to the ability of different cell types to appropriately respond to molecular signals in their particular microenvironment.

For the pulmonary fibroblasts and epithelial cells used in this study, the cellular microenvironments are quite different. The epithelial cells form a tight barrier of cell-to-cell contacts in a layer (epithelium) that lines the pulmonary airspace. There is minimum extracellular matrix around the epithelial cells except at the basal surface where they juxtapose a thin layer called the basal lamina. By contrast, the fibroblasts exist in isolation from one another in a generous layer of extracellular matrix and fibrous polymers that forms the connective tissue support of the epithelium [60]. As a result of these unique microenvironments, distinctly different biological responses are required from the resident cells. Because the epithelial cells are positioned at the forefront of the airway, their major function is to defend the lung by actions that include providing a barrier and clearance mechanism for environmental agents, modulating the inflammatory response, and regulating cellular activities in response to injury [61]. The fibroblasts, however, embedded within the interior of the tissue, assiduously maintain the integrity of the structure by producing the components of the extracellular matrix (e.g., collagens, elastin, fibronectin, and proteoglycans) and when required, migrate to sites of injury to proliferate and produce large amounts of matrix [60], [62].

Considering the diverse biological functions of pulmonary fibroblasts and epithelial cells, it is not surprising that a different structural organization would be predicted for the cell-surface HS chains of these two cell types. Since the epithelial cells are the first line of defense against injury caused by excessive release of elastase by neutrophils, they may have need for a more potent HS structure for binding and inhibiting this protease as a means to restrict its action to sites of injury or infection. Their HS structure may be more condensed (higher frequency of highly sulfated domains) because of the shorter range of operation among the closely packed epithelial cells. On the other hand, the fibroblasts exist as a sparse population in the extracellular matrix where distances are considerable. An HS structure that is more spread out (lower frequency of highly sulfated domains) may be more practical for these longer range interactions. Since the extracellular matrix also contains a convenient source of HS proteoglycans, excessive elastase activity within the matrix may be more readily addressed by extracellular HS chains, either as intact proteoglycans or as fragments released by injury. Consequently, the HS chains on the surface of the fibroblasts may have less need to be as effective in inhibiting elastase as their counterparts on the epithelial cells.

The ability to read and interpret the patterns of HS chains will have far-reaching implications for understanding the biological function of these complex glycans. The analysis of these patterns can be handled in many different ways, and as illustrated with the computer-generated chains of this study, the results can reveal varied aspects of the same chain depending on the chosen method. If the emphasis is placed on the macro organization of the chain, the calculation of the average domain size and the Fourier power spectrum are reasonable techniques for characterizing the overall domain pattern for a group of chains. However, the model generates unique chains, and although the general pattern gives a sense of the properties and potential activity of the population of chains as a whole, there is no single chain pattern that exactly matches the average chain pattern (compare Figures 3B and 4D). The existence of individual chains with unique sequences provides an opportunity to evaluate the relative density of rare patterns within the population of chains or to search for distinct local patterns within each chain. Thus, differences in the biological activities of various HS chain populations, including HS isolated from diseased and nondiseased tissues, can be correlated to differences in either the overall domain organization or the presence of specific structural motifs within the population.

Even though sequence analysis was not the impetus behind developing this computational model, it appears that it may be a powerful byproduct of the overall process. In fact, a system that can integrate the computational model with the current analytical sequencing technology may have the potential to actually “sequence” entire biologically active HS chains. The strategy behind such a system would be to use a sequencing technique to explicitly define the major oligosaccharides (ten or less sugars) from the partial degradation of the chain by one or more schemes. These fully sequenced chain fragments would then be input as a set of constraints for the model. As each chain is generated by the program, the simulated sequence would be searched for matches with the real fragments. Chains would be ranked as a function of the number of matches, and the top-scoring chain or chains would represent the best solution for the sequence of the real chain.

Although future work will focus on refining the computational model, there are two aspects of its basic design that should be emphasized. On a practical level, the first and perhaps more important factor is that the model does not require extraordinary means to achieve results. The experimental data are fairly straightforward to obtain by standard laboratory methods, and the computer program is executable on a personal computer. The second and less obvious factor is that the model has a modular structure. This allows for great flexibility in modifying specific parts of the model, such as the rules for chain position or lyase digestion, or in adding new parts, such as the generation of chains from a normal distribution of lengths. Moreover, because of this modular structure, the model is rather broad in application and can be tailored to other glycosaminoglycans or enzymes, such as chondroitin sulfate and associated chondroitin lyases.

While basic information is sufficient for operation of the model, it is apparent that the more complete these data are, the more closely the predicted chains will represent the real chains. For example, instead of using estimates of the glucuronic acid/iduronic acid ratio from the literature, improved values can be determined by comparing data from chemical and enzymatic degradations of the actual sample [36]. As another example, if the molecular weight distribution of the sample is obtained, an average chain length or a distribution of chain lengths can be defined, replacing the generation of chains over an arbitrary range of lengths with a more realistic representation of the sample [63].

An overriding issue for any simulation is how well the predictions agree with the real system. Confidence in the predicted results can only be established through model validation, and future work will focus on addressing this critical element [64]. Although there is insufficient knowledge on the domain organization of HS chains for direct comparison, an indirect method can be used to apply the model to experimental data from HPLC profiling that identify both the disaccharide and oligosaccharide products after selective heparin lyase digestion. The presence of substantial disagreement may suggest refinements to the internal rules for chain synthesis or enzyme degradation that will bring the model predictions closer to reality. A high level of agreement between the predicted results and the data will establish credibility for the model.

The true value of this model rests on whether it answers the question that prompted its development; namely, can knowing the domain organization of HS chains increase understanding of HS function. For example, activities of HS samples such as protein binding, enzyme inhibition, and cell regulation can be measured and related to domain structure. Toward this end, evaluation of the elastase inhibitory potential of the HS samples used in this study has indicated differences that might relate to altered domain organization. These differences may also have physiological implications regarding the particular role of these HS populations in their cell type of origin. For instance, the ability of HS from lung epithelial cells to inhibit elastase activity may contribute to the normal control of tissue damage at sites of inflammation where neutrophil elastase has been shown to be involved. As the model is refined and applied in conjunction with additional functional measurements with a wide range of HS samples, there are reasonable expectations that new mechanisms for the activity of HS and proteoglycans will be revealed.

The conceptual framework for an innovative computational approach to predict patterns of domain organization within a population of HS chains is presented. This model will give investigators the ability to consider high-level chain organization in understanding HS-protein interactions. The approach described here will likely provide the basis for the development of a new class of tools to probe for structure-function relationships in glycosaminoglycans that may ultimately be used to design selective drugs that target GAG-protein interactions associated with disease.
